# Muscarinic acetylcholine receptor activation prevents disinhibition-mediated LTP in the hippocampus

**DOI:** 10.3389/fncel.2013.00016

**Published:** 2013-02-28

**Authors:** Petri Takkala, Melanie A. Woodin

**Affiliations:** Department of Cell and Systems Biology, University of TorontoToronto, ON, Canada

**Keywords:** acetylcholine, neuromodulation, STDP, LTP, IPSP, hippocampus, CA1, disinhibition

## Abstract

Disinhibition-mediated long-term potentiation (LTP) in the CA1 region of the hippocampus involves GABAergic synaptic plasticity at feedforward inhibitory inputs, resulting in the reduced shunting of glutamatergic excitatory currents. The GABAergic plasticity which underlies disinhibition-mediated LTP results from a Ca^2+^-dependent decrease in the activity of the K^+^–Cl^−^ cotransporter (KCC2), depolarizing the reversal potential for GABA_A_ receptor-mediated currents (E_GABA_), thereby attenuating inhibition. Muscarinic acetylcholine receptor (mAChR) activation has previously been shown to regulate classic glutamatergic LTP, modulate intracellular [Ca^2+^] and signaling, and facilitate the excitability of GABAergic interneurons in the CA1. Based on these effects, and the ability of mAChR activation to regulate CA1 pyramidal neuron KCC2 expression, we proposed that mAChR activation would modulate disinhibition-mediated LTP. To test this prediction, we made whole cell recordings from CA1 pyramidal neurons in hippocampal slices. Disinhibition-mediated LTP was induced using a spike timing-dependent plasticity (STDP) protocol, which involved coincident pre-synaptic stimulation and post-synaptic current injection (at 5 Hz for 60 s). We found that mAChR activation via carbachol (CCh) prevented the induction of disinhibition-mediated LTP. Moreover, in the presence of CCh, E_GABA_ failed to depolarize following plasticity induction. Lastly, we recorded the paired-pulse ratio (PPR) during the induction of disinhibition-mediated LTP and found that in the presence of CCh, plasticity induction induced a significant paired-pulse depression. This suggests that pre-synaptic mAChR activation may prevent the post-synaptic expression of disinhibition-mediated LTP.

## Introduction

Long-term potentiation (LTP) is mostly studied at glutamatergic synapses onto pyramidal neurons, and is the leading cellular model of learning and memory (Malinow et al., 2000; Malenka, 2003; Lynch, 2004). This classic glutamatergic LTP depends on NMDA receptor activation, and results from AMPA receptor phosphorylation and an increase in their post-synaptic membrane expression. A novel form of LTP in the hippocampus, termed disinhibition-mediated LTP, which results from the synaptic plasticity of inhibitory GABAergic synapses has recently been demonstrated (Ormond and Woodin, 2009, 2011). When coincident pre- and post-synaptic activity induce inhibitory GABAergic plasticity at feedforward inhibitory inputs (Woodin et al., 2003), the net result is a reduced shunting of excitatory currents onto pyramidal neurons. The effect of disinhibition-mediated LTP is a long-term, synapse-specific (Ormond and Woodin, 2011) increase in the amplitude of Schaffer collateral-mediated post-synaptic potentials (PSPs).

During learning and memory processing, neuronal circuits in the hippocampus are modified by neuromodulators that regulate synaptic plasticity (Parent and Baxter, 2004; Giocomo and Hasselmo, 2007). How these neuromodulators regulate disinhibition-mediated LTP, and in turn how disinhibition-mediated LTP regulates the output of the hippocampus is not known. The objective of the present study was to determine the role of cholinergic neuromodulation in disinhibition-mediated LTP.

Hippocampal function is modulated by endogenous acetylcholine (ACh), which is released primarily from cholinergic fibers from the medial septum. Lesion of the medial septum removes the hippocampal cholinergic innervation and induces pronounced memory defects (Hagan et al., 1988; Giocomo and Hasselmo, 2007). It is also well-known that degeneration of the medial septum contributes to the cognitive deficits of Alzheimer's disease (Terry and Buccafusco, 2003). The neuromodulatory effects of ACh have also been well-documented in non-diseased human and animal behavioral experiments which show that *in vivo* administration of the muscarinic antagonist scopolamine prevents memory encoding (Ghoneim and Mewaldt, 1975; Giocomo and Hasselmo, 2007). Taken together, these studies have led in part to the prevailing idea that cholinergic modulation is essential for memory encoding in the hippocampus (Hasselmo and Giocomo, 2006).

*In vitro* hippocampal slice studies have also demonstrated a pronounced role for ACh in synaptic plasticity [the cellular basis of memory encoding (Morris et al., 2003)]. ACh acts on metabotropic muscarinic acetylcholine receptor (mAChRs) and ionotropic nicotinic ACh receptors (nAChRs) to produce a variety of neuromodulatory effects in the hippocampus (Giocomo and Hasselmo, 2007). mAChR agonists facilitate the induction of classic glutamatergic LTP in the hippocampus (Burgard and Sarvey, 1990; Huerta and Lisman, 1993; Auerbach and Segal, 1996; Shimoshige et al., 1997; Shinoe et al., 2005), however, a central mechanism underlying this enhancement has not emerged. ACh agonists and mAChR activation have profound effects on GABAergic interneurons in the CA1: they depolarize their membrane potentials (Chapman and Lacaille, 1999), increase their spiking activity (Pitler and Alger, 1992), and increase inhibitory post-synaptic current (IPSC) frequency (Pitler and Alger, 1992). Moreover, there are also neuromodulatory cholinergic effects on CA1 pyramidal neurons: mAChR activation increases pyramidal neuron excitability (Markram and Segal, 1990a,b; Huerta and Lisman, 1993; Rosato-Siri et al., 2006), and causes their depolarization (Cole and Nicoll, 1984; Widmer et al., 2006); while muscarinic receptor agonists enhance NMDA currents (Markram and Segal, 1990b), and reduce the Ca^2+^-dependent K^+^-channel current and M-currents that contribute to pyramidal neuron adaptation. Taken together, the effects of ACh on both interneurons and pyramidal neurons are to enhance neuronal excitability (by depolarizing the membrane potential toward action potential threshold) and to strengthen inhibition, as seen by increases in IPSC frequency. These ACh-induced modifications of neuronal properties, coupled with the effects of ACh on synaptic transmission and plasticity, have led us to hypothesize that mAChR-activation enhances the induction of disinhibition-mediated LTP. We tested this hypothesis by making whole-cell recordings from pyramidal neurons in the CA1 region of the hippocampus.

## Materials and methods

### Hippocampal slice preparation

All experiments were conducted using brain tissue from 14 to 40 days old male C57BL/6 mice housed under standard conditions in a 12 h light/dark cycle. Mice were housed with male littermates and provided food and water *ad libitum*. Prior to dissection, one mouse was isolated from its littermates and anesthetized using isoflurane (Halocarbon Products Corporation, River Edge, NJ, USA), followed by decapitation and rapid removable of the brain, which was then placed into chilled modified artificial cerebral spinal fluid (aCSF). This modified aCSF consisted of (mM) sucrose (216), KCl (2.5), NaH_2_PO_4_ (1.25), NaHCO_3_ (25), glucose (25), ascorbic acid (0.4), CaCl_2_ (1), MgCl_2_ (2), and sodium pyruvate (3), in double distilled water (Millipore Corporation, Billerica, MA, USA); pH = 7.4, osmolality = 300 mOsm/kg. After 1 min in chilled modified aCSF, the brain was placed onto filter paper, on which the rostral 1/3 of the brain, corresponding approximately to the forebrain anterior of the corpus callosum, and the cerebellum were removed using a sterile surgical blade (Feather, Kita-ku, Osaka, JP). The remaining tissue portion was secured to a vibratome chuck by its ventral surface, and stabilized with a 4% agar block set to abut the caudal surface of the brain. Both the tissue and agar block were submerged in chilled modified aCSF bubbled with carbogen (95% O_2_/5% CO_2_). The chuck was surrounded with ice for the duration of the slicing process. Horizontal slices were cut using a Vibratome 1000 Plus tissue sectioning system (Vibratome Company, St. Louis, MO, USA). Slices were made to a thickness of 375 μm. Slices were maintained in an interface chamber for at least 60 min at room temperature, in which Earle's Balanced Salt Solution (EBSS) (Gibco-Life Technologies, Grand Island, NY, USA) was supplemented with (mM) CaCl_2_ (1) and MgCl_2_ (3), and bubbled with 95% O_2_/5% CO_2_ carbogen. Experiments were conducted with tissue maintained in aCSF which consisted of (mM) NaCl (125), KCl (2.5), NaH_2_PO_4_ (1.25), NaHCO_3_ (25), glucose (25), CaCl_2_ (2), and MgCl_2_ (1), in double distilled water (Millipore Corporation, Billerica, MA, USA); pH = 7.4, osmolality = 300 mOsm/kg. This aCSF was bubbled with 95% O_2_/5% CO_2_ carbogen and maintained at 38°C prior to each experiment. Brain slices were longitudinally cut into hemisections; one hemisection was submerged and anchored in a slice chamber during each recording.

All experiments were performed in accordance with procedures outlined by the Canadian Council of Animal Care, and were approved by the University of Toronto Animal Care Committee.

### Electrophysiology

Experiments were performed using an Olympus BX51WI upright microscope (Olympus Canada Inc., Richmond Hill, ON, Canada). Whole-cell patch clamp recordings were made from putatively identified CA1 pyramidal neurons in aCSF flowing at 2 ml/min. Bath aCSF perfusion temperature was monitored and maintained at 37.4°C using an in-line solution heater (Warner Instruments, Hamden, CT, USA) controlled by a TC-344B Dual Automatic Temperature Controller (Warner Instruments, Hamden, CT, USA). Micropipettes were made from thin-walled borosilicate glass capillaries (TW-150F; World Precision Instruments Inc., Sarasota, FL, USA) to a resistance of 4–6 MΩ using a P-87 Flaming/Brown micropipette puller (Sutter Instrument Co., Novato, CA, USA). Micropipettes, containing a Ag/AgCl electrode, were filled with (mM) potassium gluconate (130), KCl (10), HEPES (10), EGTA (0.2), ATP (4), GTP (0.3), phosphocreatine (10); pH = 7.4, osmolality = 300 mOsm/kg. Signals were recorded using a Digidata 1322A Data Acquisition System, and Multiclamp 700B Microelectrode Amplifier controlled with Multiclamp 700B Commander software and pClamp 9.2 software (Axon Instruments Inc., Union City, CA, USA). Signal sampling was done at 200 μs intervals, and was low-pass filtered to 10 kHz. The seal leak current (I_leak_) was monitored throughout each experiment; patch-clamp recordings were abandoned if the I_leak_ exceeded 100 pA.

Extracellular stimulation of Schaffer collateral axons was applied through a micropipette containing a Ag/AgCl wire and filled with aCSF. This micropipette stimulating electrode was positioned within the stratum radiatum between the CA3 and CA1 subregions of the hippocampus, at a depth of approximately 2–3 cell layers in order to stimulate Schaffer collateral axons upon command.

Stimulus intensity was controlled using an A.M.P.I. ISO-FLEX stimulus isolator (IBIS Instrumentation Canada Inc., Ottawa, ON, CA), whereas stimulus duration and frequency were controlled using Multiclamp 700B Commander software and pClamp 9.2 software (Axon Instruments Inc., Union City, CA, USA).

### Measurement of E_GABA_ and E_rev_

E_GABA_ recordings were performed in the presence of CNQX (10 μM) to block AMPA-mediated synaptic transmission. PSPs were evoked through the extracellular stimulation of interneurons during a post-synaptic current clamp step protocol. Current clamp steps ranged from −100 to 50 pA, with a 25 pA step interval, and were applied for 600 ms while recordings were low-pass filtered at 10 kHz. Two hundred microseconds after the current clamp step was initiated, a 150 μA, 2 ms extracellular stimulus was triggered. The PSP amplitude was recorded at each current step and was plotted relative to the preceding membrane potential. This data was used to generate PSP-membrane potential plots; a simple linear regression of these plots was then used to determine E_GABA_, which was taken as the intercept of this line with the abscissa. E_rev_ is the reversal potential of mixed PSPs comprised of a summated EPSP and IPSP. Thus, E_rev_ was determined in the same manner as E_GABA_, however, AMPA receptors were not antagonized.

### Plasticity induction

Disinhibition-mediated LTP was induced in current clamp mode by pairing extracellular stimulation of pre-synaptic inputs with post-synaptic current injection (1 nA for 10 ms) at a frequency of 5 Hz for 1 min, as previously described (Ormond and Woodin, 2009, 2011). During this plasticity induction protocol the post-synaptic neuron normally fired two action potentials per pairing. The delay between pre-synaptic stimulation and post-synaptic spiking was 5–10 ms.

### Plasticity analysis

Plasticity expression was measured as a change in the magnitude of PSPs in CA1 pyramidal neurons in response to orthodromic Schaffer collateral stimulation. Disinhibition-mediated LTP was taken to consist of an increase in the PSP amplitude as well as a depolarization of E_rev_, as reported previously (Ormond and Woodin, 2009, 2011). However, mAChR-activation has been reported to depolarize pyramidal neurons (Markram and Segal, 1990a,b; Huerta and Lisman, 1993), which we also consistently observed (data not shown). Due to the depolarization of the resting membrane potential (RMP) during CCh application, we could not plot the absolute PSP amplitude. The magnitude of the PSP amplitude results from the synaptic conductance and the driving force (DF) through synaptic receptors. The DF was taken to be the difference between the RMP and E_rev_. The PSP amplitude was divided by the DF, and normalized to the average of the baseline value from the 5 min preceding plasticity induction.

### Calculation of the PPR

The paired-pulse ratio (PPR) was recorded by stimulating two PSPs with a 180 ms inter-pulse interval, repeated at 0.05 Hz for 5 min. The PPR was then calculated as the ratio of the second PSP amplitude to the first PSP amplitude (PSP = PSP_2_/PSP_1_).

### Chemicals

6-cyano-7-nitroquinoxaline-2,3-dione (CNQX) and carbachol (CCh) were purchased from Sigma-Aldrich (Sigma-Aldrich Co., St. Louis, MO, USA).

### Data analysis and statistics

**Figure 1:**
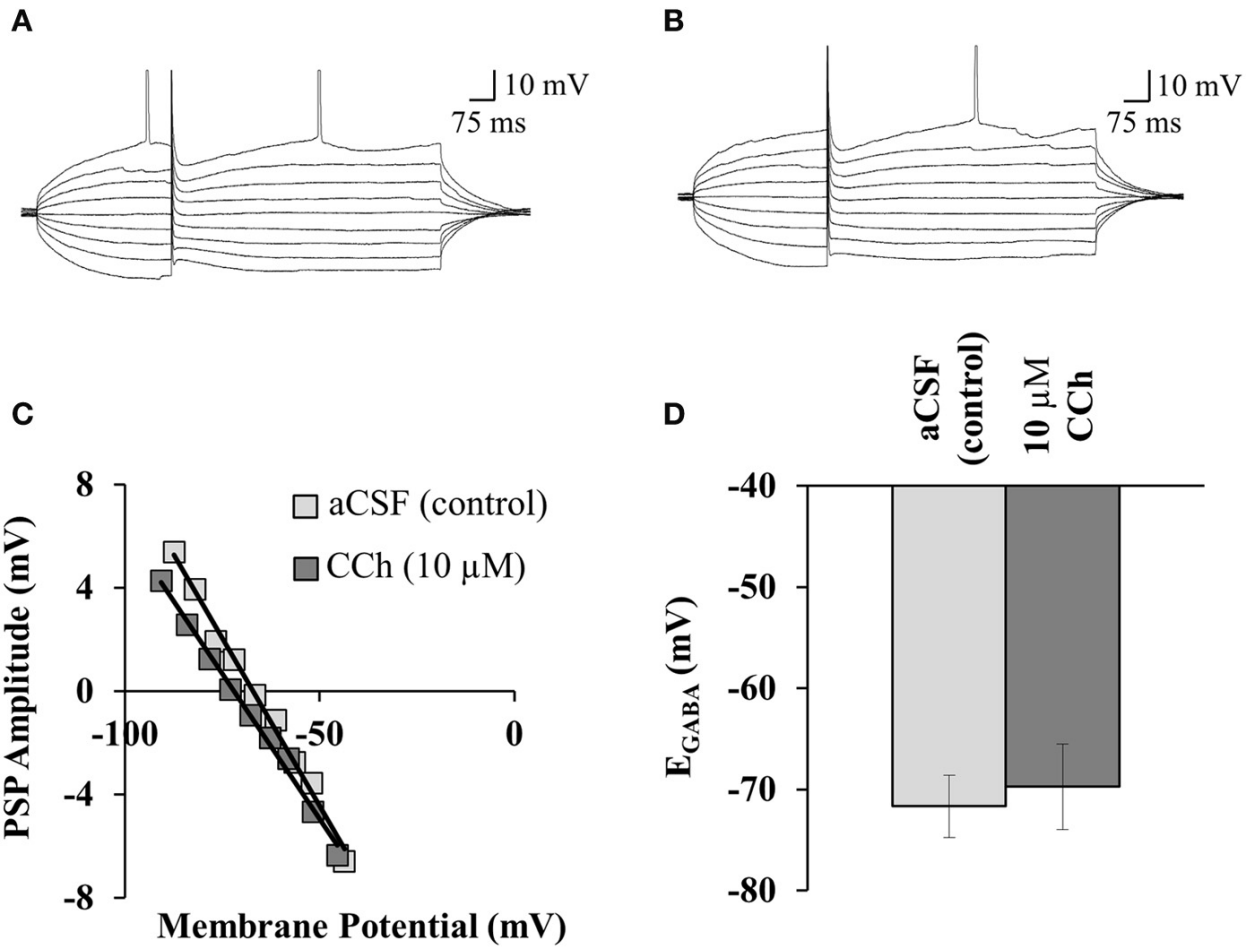
**mAChR activation does not alter E_GABA_.** Sample traces show the current clamp protocol used to record E_GABA_ after **(A)** 10 min of 10 μM CNQX, and **(B)** 10 min after the addition of 10 μM CCh in the same neuron. The mean resting membrane potential is −66.29 ± 0.31 mV in 10 μM CNQX, and −67.67 ± 0.28 mV with the addition of 10 μM CCh. **(C)** Sample PSP amplitude vs. membrane potential plots for a control neuron, and a neuron in 10 μM CCh. E_GABA_ was recorded in 10 μM CNQX, and was taken as the membrane potential where the PSP amplitude was zero (where the linear trend line intersects with the *x*-axis). **(D)** Summary of all experiments similar to **(C)**. Average E_GABA_ for control (*n* = 7) and CCh perfusion (*n* = 7). Error bars represent ± one SEM.

**Figure 2:**
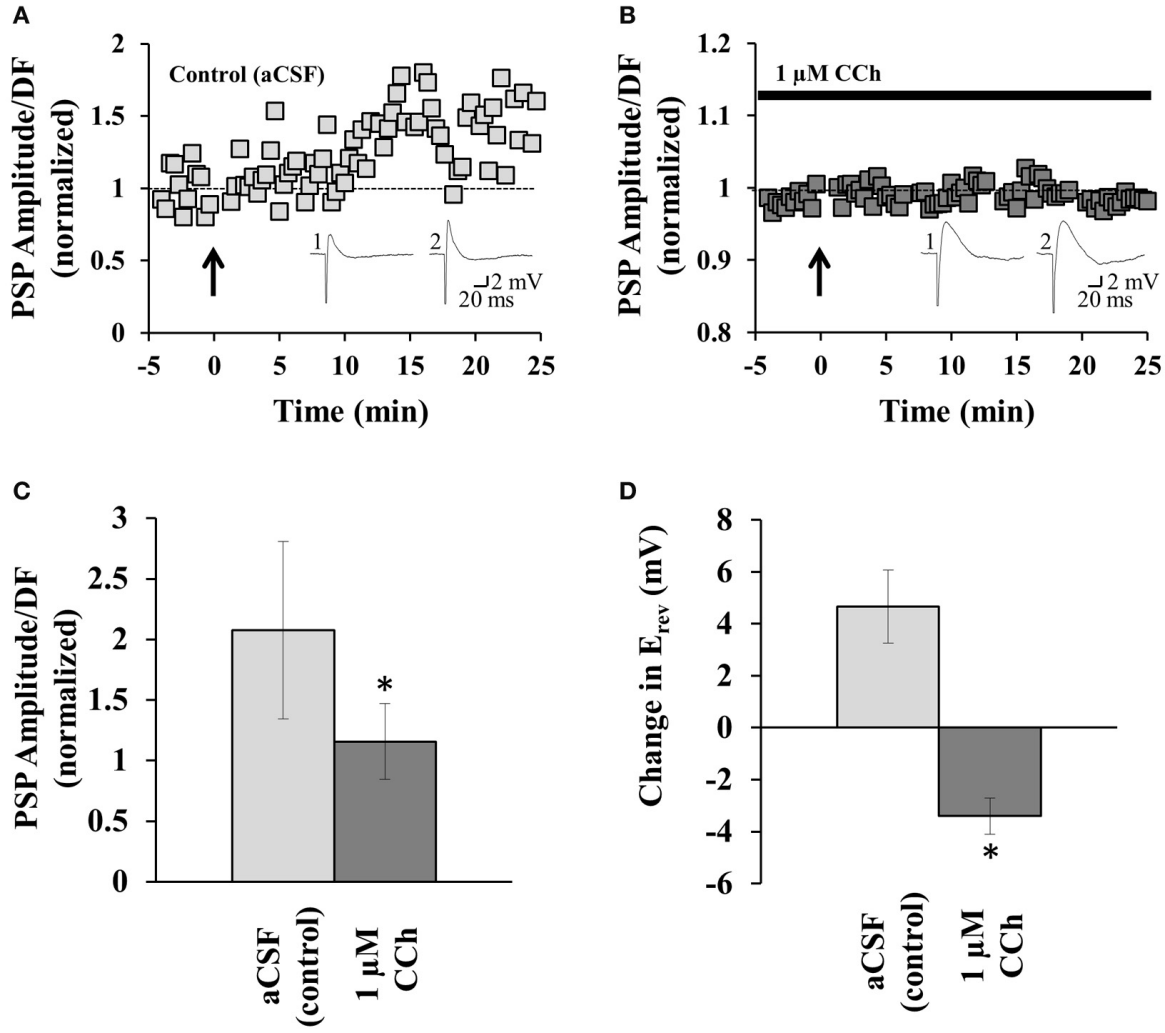
**mAChR activation prevents disinhibition-mediated LTP. (A)** Example recording from one neuron before and after the induction of disinhibition-mediated LTP (induced at arrow; coincident pre- and post-synaptic activity at 5 Hz for 1 min). PSP amplitude/driving force (DF) values are normalized to the pre-induction baseline (see the Plasticity Analysis section in Experimental Procedures for details on normalization). Insets: sample PSP amplitude recordings before plasticity induction (1), and from the end of the recording period (2). **(B)** Similar example recording to that in **(A)**, but for a neuron perfused with 1 μM CCh. **(C)** Summary of all experiments similar to those in **(A)** (*n* = 9) and **(B)** (*n* = 4). **(D)** Summary of the change in E_rev_ for neurons in aCSF or CCh. ^*^indicates significance (*p* < 0.001). Error bars represent ± one SEM. Dashed lines indicate normalized pre-induction amplitude.

**Figure 3:**
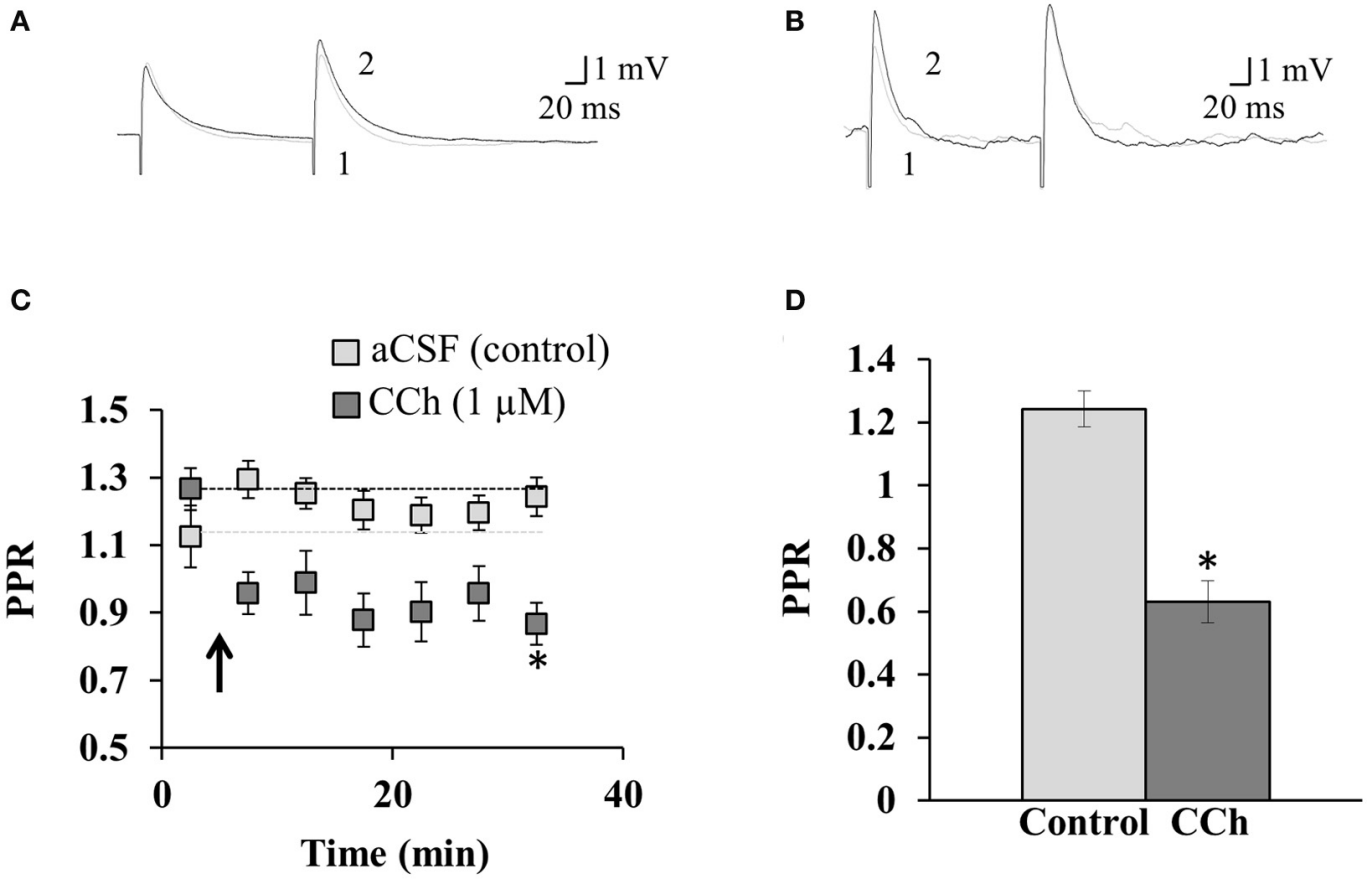
**The mAChR-mediated prevention of disinhibition-mediated LTP is accompanied by a decrease in the PPR.** Sample traces show the paired-pulse protocol in **(A)** aCSF and **(B)** 1 μM CCh. Pre-induction recordings of the PPR are shown in gray (1), overlain with post-induction PPR recordings in black (2) from the same neuron. **(C)** The average PPR before and after the induction of disinhibition-mediated LTP (induced at arrow; coincident pre- and post-synaptic activity at 5 Hz for 1 min), for neurons perfused with aCSF or CCh. ^*^Indicates significance (*p* = 0.002). Dashed lines indicate normalized pre-induction amplitude for each condition. **(D)** Summary of the PPR ratio in the last 5 min of the recording for neurons perfused with aCSF (*n* = 9) or CCh (*n* = 4). ^*^Indicates significance (*p* < 0.001). Error bars represent ± one SEM.

Data were acquired using Axon Instruments Clampex 9 software, and analyzed using Axon Instruments Clampfit (Axon Instruments Inc., Union City, CA, USA). Results are expressed as mean ± standard error of the mean (SEM). All statistical tests were performed in SigmaStat (Systat Software, San Jose, CA, USA). Statistical analyses were performed using the following tests: Figure 1D, Student's *t*-test; Figures 2A,B, Two-Way ANOVA followed by Student's *t*-test; Figures 2C,D, Student's *t*-test; Figure 3C, Two-Way ANOVA, Figure 3D, Student's *t*-test.

## Results

### mAChR activation does not regulate E_GABA_

Our main objective was to determine whether mAChR activation modulates disinhibition-mediated LTP in the CA1 region of the hippocampus. However, because the mechanism underlying disinhibition-mediated LTP is a depolarization of the reversal potential for PSPs (E_rev_), which results from a depolarization of the reversal potential for inhibitory GABA_A_ receptor-mediated currents (E_GABA_) (Woodin et al., 2003; Ormond and Woodin, 2009, 2011; Lamsa et al., 2010), we first had to determine whether mAChR activation alone (prior to plasticity induction) modulates E_GABA_. This was especially important in light of a recent report demonstrating that prolonged mAChR activation promotes KCC2 degradation (Lee et al., 2010), which could presumably depolarize E_GABA_.

To determine whether mAChR activation regulates E_GABA_, we stimulated GABA release from interneurons close to the CA1 stratum pyramidale while recording GABA_A_ receptor currents in putatively identified CA1 pyramidal neurons. GABA_A_ currents were isolated by pharmacologically inhibiting AMPA receptors with CNQX (10 μM); NMDA receptors were not inhibited because they are required for the induction of disinhibition-mediated LTP. GABA_A_ receptor currents were evoked during depolarizing current steps (Figures 1A,B), and the current amplitude was plotted against the membrane potential; the intercept of this curve with the abscissa was taken as E_GABA_ (Figure 1C). In standard aCSF (control), the mean E_GABA_ was −71.67 ± 3.07 mV (*n* = 7; Figure 1D). When slices were perfused with the mAChR agonist (CCh; 10 μM), there was no significant difference in E_GABA_ compared to slices perfused with aCSF (−69.74 ± 4.21 mV; *n* = 7; *p* = 0.72, Student's *t*-test; Figure 1D). Thus, mAChR activation does not acutely regulate E_GABA_ in hippocampal neurons.

### mAChR activation prevents disinhibition-mediated LTP and the depolarization of E_rev_

mAChR activation increases the spiking activity of GABAergic interneurons in the CA1 (Pitler and Alger, 1992), and increases the excitability of pyramidal neurons (Cole and Nicoll, 1984; Markram and Segal, 1990a,b; Huerta and Lisman, 1993). Since disinhibition-mediated LTP is induced by correlated pre- and post-synaptic activity (Ormond and Woodin, 2009), we predicted that the mAChR-mediated increase in interneuron and pyramidal neuron spiking would facilitate plasticity induction. To test this hypothesis we induced disinhibition-mediated LTP in the absence and presence of CCh. Disinhibition-mediated LTP was induced by pairing pre-synaptic stimulation with post-synaptic spiking at 5 Hz for 1 min. Figure 2A shows an example induction of disinhibition-mediated LTP (control; aCSF perfusion); when all experiments similar to this example were summarized there was a significant increase in the PSP amplitude (*p* < 0.001, *p* value is reported for last 5 min of the recording compared to the 5 min pre-induction baseline, Student's *t*-test; *n* = 9). However, when these experiments were repeated in the presence of continuous CCh perfusion (1 μM), disinhibition-mediated LTP failed to be induced (*p* = 0.992; *n* = 4), as seen in the example trace in Figure 2B. When we compared the recordings from control and CCh experiments we found that there was a significant difference in PSP amplitude (comparing the last 5 min of each recording (20–25 min bin), *p* < 0.001, Student's *t*-test; Figure 2C). Thus, in contrast to our prediction that mAChR activation would facilitate the induction of disinhibition-mediated LTP, mAChR activation not only failed to facilitate plasticity, it prevented plasticity induction.

As explained above, disinhibition-mediated LTP results from a depolarization of the E_rev_. If mAChR activation prevents plasticity induction, then we should fail to see a depolarization of E_rev_ in the presence of CCh, if the plasticity was to be expressed post-synaptically (Ormond and Woodin, 2009, 2011). To test this prediction, we compared the change in the E_rev_ at the end of the plasticity experiment to the pre-induction baseline. In the control (aCSF) experiment there was a 4.66 ± 1.41 mV depolarization in the E_rev_ (*n* = 9; *p* = 0.046, Student's *t*-test); which was consistent with previous findings (Ormond and Woodin, 2009, 2011). However, in the presence of CCh there was a 3.39 ± 0.69 mV hyperpolarization of E_rev_ (*n* = 4; *p* = 0.04, Student's *t*-test). The change in E_rev_ between control and CCh was significant (*p* < 0.001, Student's *t*-test; Figure 2D). Thus, mAChR activation prevented disinhibition-mediated LTP by preventing the post-synaptic E_rev_ depolarization.

### The mAChR-induced prevention of disinhibition-mediated LTP is accompanied by paired-pulse depression

We next asked whether the mechanism by which mAChR activation prevents disinhibition-mediated LTP might reside pre-synaptically. To address this, we recorded the PPR (Figures 3A,B), which is commonly used as a procedure to identify changes in the probability of transmitter release, and thus suggests whether the mechanism of plasticity arises pre- or post-synaptically (Schulz et al., 1994, 1995). The PPR prior to the induction of disinhibition-mediated LTP (in aCSF) was 1.13 ± 0.09 mV (*n* = 9, Figure 3C). Furthermore, the induction of disinhibition-mediated LTP in aCSF did not result in a significant change in the PPR (*p* = 0.233, One-Way ANOVA, *n* = 9), which is consistent with this form of plasticity resulting from a post-synaptic change (Ormond and Woodin, 2009, 2011). During the last 5 min of the recording, the PPR (in aCSF) was 1.24 ± 0.06 mV (*n* = 9). When we examined the PPR following plasticity induction in the presence of 1 μM CCh, we found that the PPR decreased from 1.27 ± 0.04 mV (*n* = 4) before induction, to 0.86 ± 0.04 mV (*n* = 4) during the last 5 min. The inclusion of CCh lead to a significant depression of the PPR relative to the control experiment (*p* = 0.002, Two-Way ANOVA, Figure 3C). Furthermore, the PPR was significantly depressed relative to the control in the final bin of each experiment (*p* < 0.001, Student's *t*-test, Figure 3D). This paired-pulse depression, which began immediately following the plasticity induction protocol, suggests that mAChR activation acts via a pre-synaptic mechanism to prevent the post-synaptic expression of disinhibition-mediated LTP.

## Discussion

Disinhibition-mediated LTP results from inhibitory synaptic plasticity of feedforward inhibitory inputs onto pyramidal neurons (Ormond and Woodin, 2009, 2011). The mechanism underlying inhibitory synaptic plasticity is a post-synaptic Ca^2+^-mediated decrease in KCC2 function, which depolarizes E_GABA_, essentially weakening synaptic inhibition (Woodin et al., 2003; Saraga et al., 2008; Lamsa et al., 2010). It has previously been shown that ACh, acting on M_1_ receptors, potentiates Ca^2+^ transients via G-protein coupled signal transduction, and through second messengers is known to increase the intracellular calcium concentration from intracellular stores (Kirkwood et al., 1991; Gulledge and Stuart, 2005). This suggests that a G-protein coupled mAChR-mediated [Ca^2+^] increase can modulate E_GABA_ by altering the functional expression of KCC2. There is growing evidence in the literature that neuromodulators may regulate E_GABA_. For example, Lee et al. (2010) demonstrated that prolonged activation of mAChRs on cultured hippocampal neurons enhances lysozomal degradation of KCC2, which would presumably depolarize E_GABA_. However, in the present study we found that mAChR activation did not alter E_GABA_. The discrepancy in these results likely arises from the high concentration of CCh (100 μM) and prolonged use (120 min) of CCh in the Lee et al. study, compared to the present procedures (10 μM, 15–30 min).

When disinhibition-mediated LTP was induced in the presence of mAChR activation, we observed a rapid paired-pulse depression immediately following plasticity induction. Short-term depression is attributed to a decrease in pre-synaptic neurotransmitter release, often due to a depletion of releasable vesicles (Liley and North, 1953; Dobrunz and Stevens, 1997; Bellingham and Walmsley, 1999). If this is the case in our present experiments, it would suggest that despite the ability of mAChR-activation to increase interneuron spiking (Pitler and Alger, 1992), there was a reduction in GABA release which could account for the inability of disinhibition-mediated LTP to be induced. The possible reduction in GABA release could result from a direct action of mAChR signaling pathways on the release of GABA containing synaptic vesicles. This possibility is supported by the finding that when the M_2_ mAChR is knocked-out, there is an increase in the strength of GABAergic inhibition, which accounts for an impairment in the expression of classic glutamatergic LTP (Shimoshige et al., 1997; Seeger et al., 2004). Further investigation into this mechanism should consider whether the potential CCh-induced decrease in GABA release occurs during plasticity induction, and whether M_2_ receptor activation is sufficient to inhibit the expression of disinhibition-mediated LTP.

Alternatively, paired-pulse depression and the prevention of disinhibition-mediated LTP in the presence of CCh may be due to a mAChR desensitization; implicating a post-synaptic locus of neuromodulation. Previous studies have demonstrated a desensitization of mAChR-mediated signal transduction by CCh in hippocampal neurons (Lenox et al., 1988; Pontzer and Crews, 1990). Notably, Adams et al. (2004) showed an inhibition of LTP induction by a spike-pairing stimulation at the Schaffer collateral-CA1 synapse, and speculated that the modulatory action of mAChR activation on LTP at glutamatergic synapses results from receptor desensitization. However, Pontzer and Crews (1990) demonstrated that the CCh-mediated desensitization of mAChR signal transduction in hippocampal neurons effectively occurs only at higher concentrations of the agonist (i.e., 5–30 μM), beyond the 1 μM concentration used in our study.

Further investigation is required in order to determine whether any of these proposed mechanisms underlie the CCh-mediated prevention of disinhibition-mediated LTP. To this end, it would be prudent to consider the known expression pattern of mAChR subtypes, and consider their relative affinity to CCh. Notably, M_2_ receptors on pre-synaptic interneuron terminals have previously been shown to suppress GABA release (Fukudome et al., 2004), and pre-synaptic M_1_ and M_2_ have been shown to suppress Schaffer collateral-CA1 synaptic potentials (Kremin et al., 2006). In addition, GABA release suppression by retrograde signaling has also been demonstrated (Ohno-Shosaku et al., 2003), therefore post-synaptic M_1_ and M_3_ receptors may act indirectly to suppress synaptic transmission. A robust pharmacological investigation, paired with the appropriate genetic tools can be used to further investigate the underlying mechanism by which CCh prevents disinhibition-mediated LTP.

In conclusion, the present study provides the first evidence that disinhibition-mediated LTP is modulated by the cholinergic neuromodulatory system. Specifically, we found that mAChR-activation prevents the induction of disinhibition-mediated LTP in CA1 pyramidal neurons. Disinhibition-mediated LTP is expressed post-synaptically via a depolarization of the E_rev_ (Ormond and Woodin, 2009). While mAChR-activation prevented the depolarization of E_rev_ post-synaptically, we also observed a paired pulse depression following plasticity induction, which suggests that the mechanism of prevention may reside pre-synaptically. In addition to providing novel evidence for the neuromodulation of disinhibition-mediated LTP, this is the first study to demonstrate that disinhibition-mediated LTP can be induced in the mouse hippocampus.

### Conflict of interest statement

The authors declare that the research was conducted in the absence of any commercial or financial relationships that could be construed as a potential conflict of interest.
